# Resting-State Functional Connectivity between Putamen and Salience Network and Childhood Body Mass Index

**DOI:** 10.3390/neurolint13010009

**Published:** 2021-03-04

**Authors:** Shervin Assari, Shanika Boyce

**Affiliations:** 1 Department of Family Medicine, Charles R. Drew University of Medicine and Science, Los Angeles, CA 90059, USA; 2 Department of Urban Public Health, Charles R. Drew University of Medicine and Science, Los Angeles, CA 90059, USA; 3 Department of Pediatrics, Charles R. Drew University of Medicine and Science, Los Angeles, CA 90059, USA; shanikaboyce@cdrewu.edu

**Keywords:** children, fMRI, brain development, population groups, putamen, functional connectivity, obesity, body mass index

## Abstract

Introduction: Although the putamen has a significant role in reward-seeking and motivated behaviors, including eating and food-seeking, minorities’ diminished returns (MDRs) suggest that individual-level risk and protective factors have weaker effects for Non-Hispanic Black than Non-Hispanic White individuals. However, limited research is available on the relevance of MDRs in terms of the role of putamen functional connectivity on body mass index (BMI). Purpose: Building on the MDRs framework and conceptualizing race and socioeconomic status (SES) indicators as social constructs, we explored racial and SES differences in the associations between putamen functional connectivity to the salience network and children’s BMI. Methods: For this cross-sectional study, we used functional magnetic resonance imaging (fMRI) data of 6473 9–10-year-old Non-Hispanic Black and Non-Hispanic White children from the Adolescent Brain Cognitive Development (ABCD) study. The primary independent variable was putamen functional connectivity to the salience network, measured by fMRI. The primary outcome was the children’s BMI. Age, sex, neighborhood income, and family structure were the covariates. Race, family structure, parental education, and household income were potential moderators. For data analysis, we used mixed-effect models in the overall sample and by race. Results: Higher right putamen functional connectivity to the salience network was associated with higher BMI in Non-Hispanic White children. The same association was missing for Non-Hispanic Black children. While there was no overall association in the pooled sample, a significant interaction was found, suggesting that the association between right putamen functional connectivity to the salience network and children’s BMI was modified by race. Compared to Non-Hispanic White children, Non-Hispanic Black children showed a weaker association between right putamen functional connectivity to the salience network and BMI. While parental education and household income did not moderate our association of interest, marital status altered the associations between putamen functional connectivity to the salience network and children’s BMI. These patterns were observed for right but not left putamen. Other/Mixed Race children also showed a pattern similar to Non-Hispanic Black children. Conclusions: The association between right putamen functional connectivity to the salience network and children’s BMI may depend on race and marital status but not parental education and household income. While right putamen functional connectivity to the salience network is associated with Non-Hispanic White children’s BMI, Non-Hispanic Black children’ BMI remains high regardless of their putamen functional connectivity to the salience network. This finding is in line with MDRs, which attributes diminished effects of individual-risk and protective factors for Non-Hispanic Black children to racism, stratification, and segregation.

## 1. Introduction

The putamen, a round brain structure located at the basal ganglia, is a significant dorsal striatum [1]. The putamen is involved in a wide range of motivated behaviors, decision making, and addiction [1]. As part of the brain reward system, putamen is a part of the brain’s dopaminergic system that influences a wide range of motivated behaviors including eating [2,3,4]. 

As an element of the brain dopaminergic system, putamen regulates the brain reward system [5]. Altered putamen structure and function predict poor decision-making, impulsivity, and high-risk behaviors, including but not limited to substance use [6]. One of the mechanisms that increases individuals’ risk of tobacco [7,8,9], drug [10,11,12], and alcohol [13,14,15] use is altered putamen function, through its association with impulsivity and reward seeking [16].

According to minorities’ diminished returns (MDRs) [17], observed across study designs, cohorts, settings, age groups, socioeconomic status (SES) indicators, and health outcomes [18,19], individual-level risk and protective factors show weaker associations with outcomes of racial and ethnic minority people compared to Non-Hispanic Whites. For example, SES shows weaker associations with brain structure and function of Non-Hispanic Blacks than Non-Hispanic Whites [20,21,22,23,24,25,26,27]. The same pattern is shown for SES effects on trauma [28], attention deficit hyperactivity disorder (ADHD) [29], suicide [27], depression [30], aggression [31], tobacco use [31,32,33], impulsivity [34], school bonding [35], school performance [36], math performance [37], attention [38], and inhibitory control [39] in Non-Hispanic Black children compared with Non-Hispanic White children. Similar results are shown in the Adolescent Brain Cognitive Development (ABCD) study [26,27,39,40], Add Health [17], the Fragile Families and Child Wellbeing Study (FFCWS) [29,34,35,41,42,43,44], Monitoring the Future (MTF) [36], the National Survey of American Life (NSAL) [30], the Flint Adolescents Study (FAS) [45], the Population Assessment of Tobacco and Health (PATH) [31], the Early Childhood Longitudinal Study (ECLS) study [46], and the Family and Community Health Study (FACHS) [47,48].

The MDRs have been shown for outcomes such as obesity [44], tobacco use [31], suicide [27], and aggression [31], which are all linked to impulsivity. In the ABCD [26,27,39], FFCWS [29,34,44], and PATH [31] studies, high SES Non-Hispanic Black children have remained at a high level of impulsivity [21,39], risk-taking [31], and reward-dependency [26,49], to a level that is unexpected given their SES. For example, Non-Hispanic Black children from high SES backgrounds showed worse than expected impulsivity [34], inhibitory control [21], fun-seeking [49], and reward responsiveness [26]. As a result, high SES Non-Hispanic Black children remain at risk of obesity, regardless of their individual level risk and protective factors [44,50,51,52]. While this literature is mainly limited to the effects of SES, with the same line of reasoning, putamen function and functional connectivity may have less salience as determinants of children’s developmental and behavioral outcomes such as body mass index (BMI) for Non-Hispanic Black youth.

In this study, we conceptualized race and SES as social rather than biological constructs [53] and explored racial and SES differences in the association between putamen functional connectivity with salience network and children’s BMI. We focused on the putamen as it has major implications for risk-taking, impulsivity, and sensation seeking, all related to children’s BMI [54,55,56,57,58]. We hypothesized that putamen functional connectivity to the salience network would be related to BMI (Hypothesis 1) [55,59,60,61,62,63]. In line with the MDRs phenomenon, we expected a weaker association between putamen functional connectivity to the salience network and BMI for Non-Hispanic Black children in comparison with Non-Hispanic White children (Hypothesis 2). Again, this is not because Non-Hispanic Blacks and Non-Hispanic Whites are biologically different but because race, as a proxy for exposure to racism, racialization, stratification, discrimination, and adversities, interferes with the likelihood that individuals secure outcomes in the presence of resources and assets [53]. As some research has shown specific lateralization of brain reward system including putamen, and as past research has shown that reward-related to food is connected to right putamen activity, we expected MDRs for right but not left putamen. Finally, as race and SES closely overlap, we tested if similar MDRs can be seen across groups in terms of parental education, household income, and family structure.

## 2. Methods

### 2.1. Design and Settings

This is a secondary analysis of existing data. Data are from the Adolescent Brain Cognitive Development (ABCD) study [64,65,66,67]. The ABCD is a landmark brain development study in the United States. Although detailed information regarding ABCD study methods, sampling, sample, measures, and imaging techniques are available [64,65,66,67,68,69], we briefly review some key aspects of the study.

### 2.2. Participants and Sampling

Participants of the ABCD study were recruited as children between ages 9 and 11 from multiple cities across the USA. Overall, participants were enrolled from 21 sites. The primary source of recruitment for the ABCD sample was U.S. school systems. The sampling protocol of the ABCD study is described in detail elsewhere [64]. This analysis was based on 6473 participants; in this analysis, participants needed to have valid data on race, ethnicity, demographics, right putamen functional connectivity to the salience network, parental marital status, and children’s BMI. Participants were included if their Monetary Incentive Delay (MID) task was available, and T1 MRI was recommended to be used in the Data Exploration and Analysis Portal (DEAP).

### 2.3. Study Variables

The study variables included right putamen functional connectivity to the salience network (independent variables); child race (moderators); child age, ethnicity, sex, and parental marital status (confounders); and child BMI (dependent variables).

#### 2.3.1. Main Outcomes

*Body Mass Index (BMI)*. The main outcomes were the children’s BMI, calculated on the basis of the measured height and weight of the children.

#### 2.3.2. Independent Variable

*Right putamen functional connectivity to the salience network.* Participants’ right putamen functional connectivity to the salience network education were a continuous variable. This variable is pre-calculated and available in the ABCD dataset. This was measured using resting functional magnetic resonance imaging (fMRI). For more information on ABCD fMRI techniques and details, please see Casey and others [69]. The ABCD imaging protocol was harmonized for three 3T scanner platforms (Siemens *Prisma*, General Electric (GE) 750, and Philips) and use of multi-channel coils capable of multiband echo planar imaging (EPI) acquisitions, using a standard adult-size coil. The scan session consisted of a fixed order of scan types that begin with a localizer, acquisition of 3D T1-weighted images, 2 runs of resting state fMRI, diffusion-weighted images, 3D T2-weighted images, 1–2 more runs of resting state fMRI (see motion detection below for when to acquire 1 versus 2 additional runs), and the task-based fMRI. Participants completed an MRI screening questionnaire for any contraindication for an MRI (e.g., braces, pacemakers, and other metal in the body including piercings, medical screw, or pins). This MR screening occurred 3 times: during initial recruitment, at scheduling, and just prior to the scan. Before the scan, participants were desensitized to the scanner environment with a simulator. The simulation occurred in dedicated mock scanners with a prerecorded scanner. A child-friendly movie was turned on as the child entered the scanner and remained on during acquisition of the localizer and 3D T1 scans, being also played during the 3D T2 and diffusion-weighted imaging acquisitions. The functional scans included 20 min of resting-state data acquired with eyes open and passive viewing of a cross-hair. One set of two 5 min runs was acquired immediately after the 3D T1 and another set was acquired after the 3D T2 scans. Real-time motion detection and correction for the structural scans were implemented by the ABCD DAIC hardware and software. A real-time head motion monitoring system called FIRMM (fMRI integrated real-time motion monitor (www.firmm.us (accessed on 1 March 2021)) [70], collaboratively developed at Washington University, St. Louis, and Oregon Health Sciences University was implemented for motion detection in resting state fMRI scans at the Siemens sites. Scan qualities were acceptable given the young age of the participants (9–10 years), length of the scan protocol (100–120 min), and that approximately 42% of the sample consisted of children who showed early signs of externalizing and internalizing symptoms and were considered at risk for substance abuse and other mental health problems [69].

#### 2.3.3. Moderators

*Race.* Children’s race was self-identified by the parents. Race was a dichotomous variable: Non-Hispanic Black vs. Non-Hispanic White (reference category).

#### 2.3.4. Confounders

Age, sex, parental marital status, and neighborhood income were included as covariates. Parents reported the child’s age, which was calculated as months between the date of birth and the study’s date. Sex of the child was a dichotomous variable that was coded 0 for males and 1 for females. Parental marital status was also a dichotomous variable, self-reported by the parent interviewed, and coded 1 vs. 0 for married and unmarried. Neighborhood median income was derived from ABCD residential history files. These data were collected from Zip codes. This variable was derived from residential history data of the ABCD and was calculated for current place of residence, regardless of duration of residence in the area. Neighborhood income was treated as a continuous measure, with higher neighborhood income being an indicator of higher area-level SES [71,72,73,74,75,76].

### 2.4. Data Analysis

We used the Data Exploration and Analysis Portal (DEAP) for data analysis. Provided by the Data Analysis and Informatics Core of the ABCD study. The DEAP uses R and provides a user-friendly online platform for multivariable analysis of the ABCD data. The DEAP platform was obtained from https://deap.nimhda.org (accessed on 1 March 2021), and ABCD data were downloaded from https://nda.nih.gov/abcd (accessed on 1 March 2021). For our univariate analysis, we reported the mean (standard deviation (SD)) and frequency (%) of our variables depending on the variable type. We also reported the results of the chi-squared test to compare Non-Hispanic White and Non-Hispanic Black children. Our regression in DEAP was based on mixed-effect models, given the fact that participants are nested to families, and families are nested to sites. The primary outcome was the children’s BMI. The independent variable was the right putamen functional connectivity to the salience network. The moderator was race. Age, sex, family marital status, and neighborhood income were covariates. Overall, four models were fitted. *Model 1* and *Model 2* were run in Non-Hispanic White and Non-Hispanic Black children, respectively. *Model 3* tested the additive effects, while covariates were in the model. This model did not include an interaction term. *Model 4* tested the interaction term between right putamen functional connectivity to the salience network and race. Before running models, we checked a wide range of assumptions, including lack of collinearity between predictors, normal distribution of our outcome, distribution of errors for our model, as well as the association between observed and theoretical quantiles of our model (Appendix A). Other than the main analysis explained above, we performed additional analysis as below. First, we tested if the same patterns held for right and left putamen. Second, we ran models with parental education, household income, and family structure as moderators. Finally, as MDRs are not specific to Non-Hispanic Blacks, we also tested the same patterns for other non-White groups. From regression coefficients, we reported beta coefficients, Standard Errors (SE), and *p*-values. *p*-values smaller than 0.05 were significant.

### 2.5. Ethical Aspect

Our secondary analysis was found by the Charles R Drew University of Medicine and Science (CDU) Institutional Review Board (IRB) to be exempt from a full IRB review (IRB number 1665000-1; Date: 10/02/2020). However, the original ABCD study underwent an Institutional Review Board (IRB) in several institutions, including but not limited to the University of California, San Diego (UCSD). The IRB in multiple institutions approved the study protocol, and all children provided assent and parents signed consent.

## 3. Results

### 3.1. Sample Descriptive Data

Table 1 shows the descriptive data, overall and by race. Our sample included 6473 children who were either 9 or 10 years old. Among the sample, 5180 (80.0%) were Non-Hispanic White, and 1293 (20.0%) were Non-Hispanic Black. As Table 1 shows, right putamen functional connectivity to the salience network was higher in Non-Hispanic White than Non-Hispanic Black children. Non-Hispanic White and Non-Hispanic Black children also differed in terms of BMI. Non-Hispanic White children had lower average BMI than Non-Hispanic Black children.

### 3.2. Model Fit

As Table 2 shows, the best fitting model was *Model 4*, which was performed in the full sample, and had the interaction term.

### 3.3. Effects in Non-Hispanic White and Non-Hispanic Black Participants

As shown in Table 3, the right putamen functional connectivity to the salience network positively associated children’s BMI in Non-Hispanic White children but not in Non-Hispanic Black children. For Non-Hispanic White children, the association was positive and significant. Although not significant, for Non-Hispanic Black children, the association was negative.

Table 3 shows the results of *Model 1* and *Model 2*. Higher right putamen functional connectivity to the salience network was associated with higher BMI in Non-Hispanic White children. The same association was missing for Non-Hispanic Black children.

### 3.4. Main Effects in the Pooled Sample

Table 4 summarizes the results of *Model 3* and *Model 4*. As shown in Table 4, right putamen functional connectivity to the salience network had an inverse association with children’s BMI. As Table 4 shows, we also found a statistically significant interaction between the effects of right putamen functional connectivity to the salience network and race on children’s BMI. This interaction suggested that the gain in terms of low BMI from higher levels of right putamen functional connectivity to the salience network is diminished for Non-Hispanic Black compared to Non-Hispanic White children.

**Figure 1:**
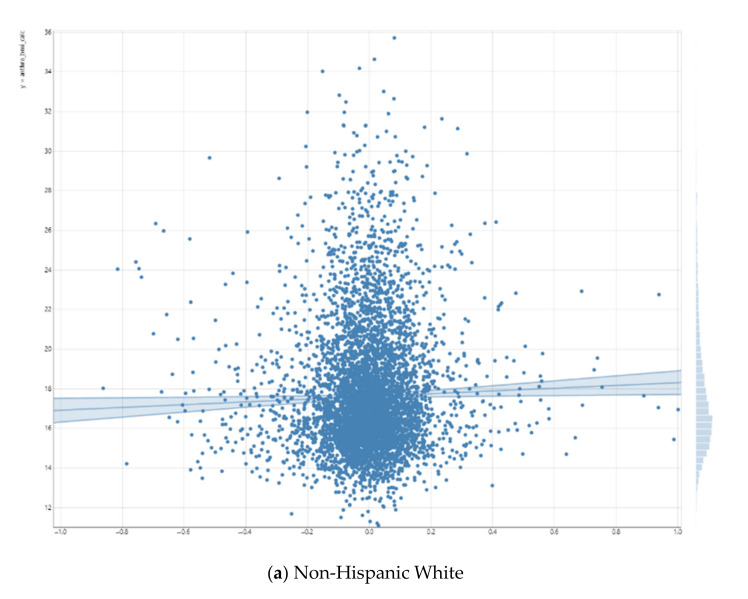

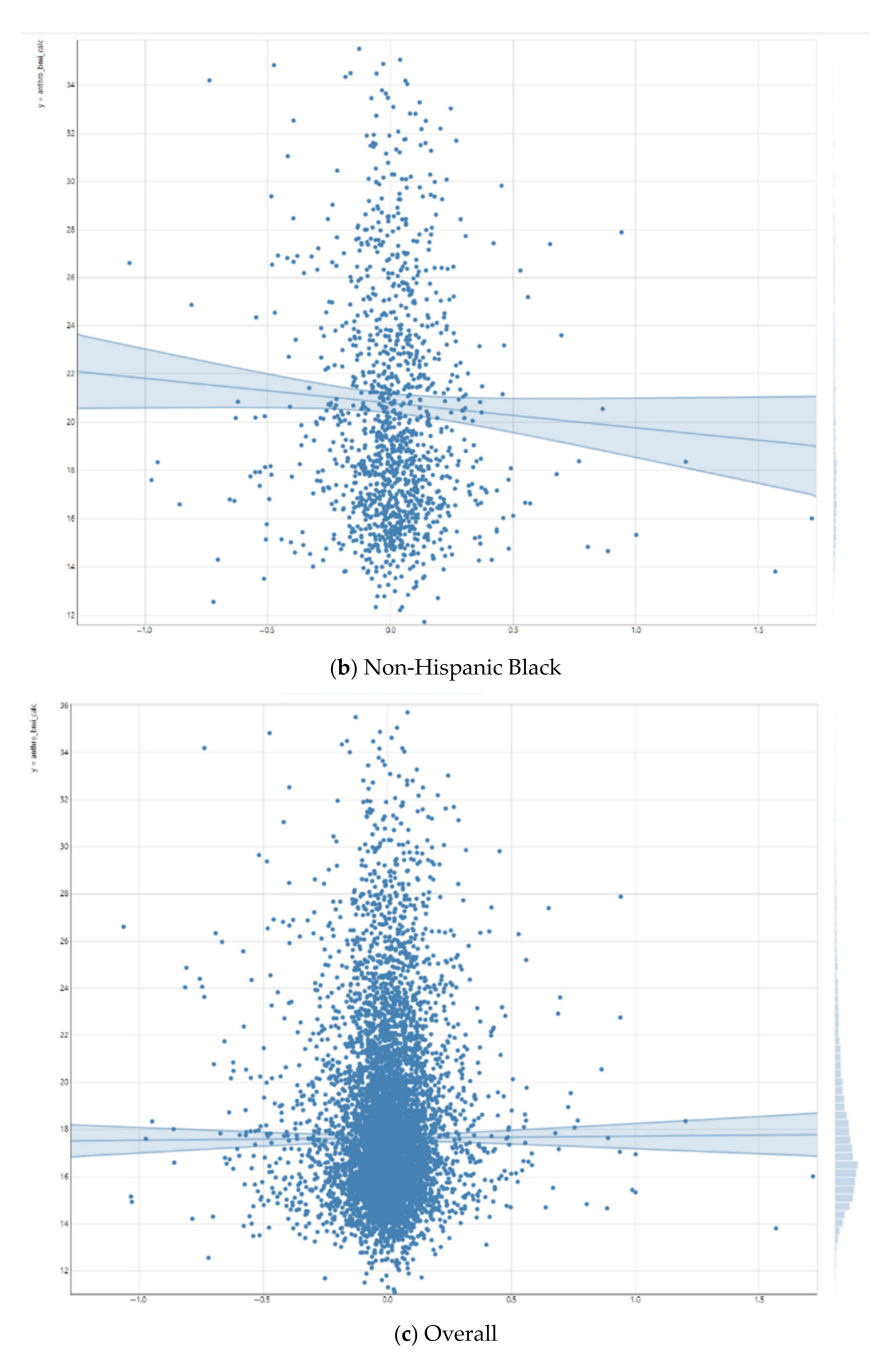

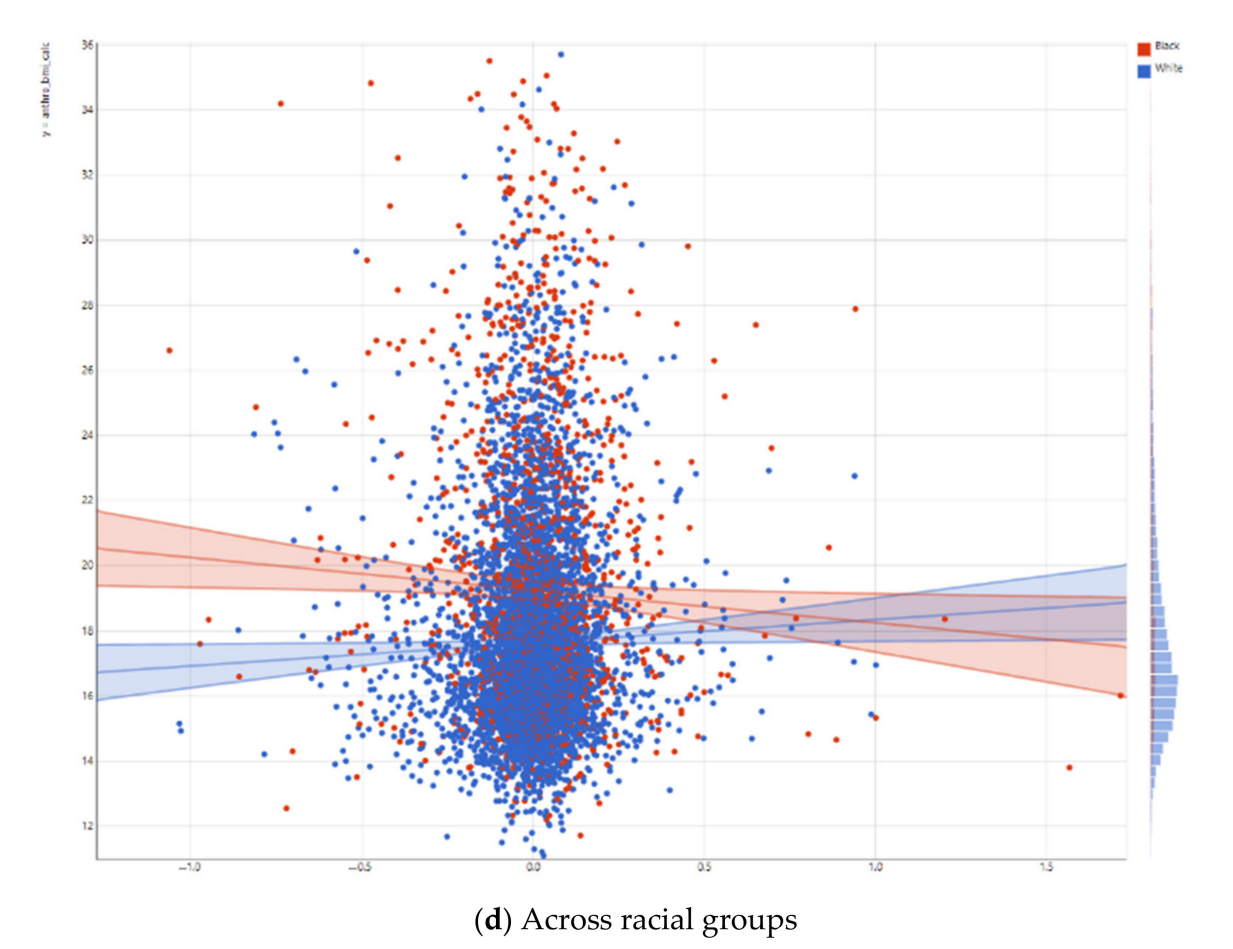
Association between right putamen functional connectivity to the salience network and children’s body mass index (BMI).

As shown in Figure 1, the right putamen functional connectivity to the salience network had a positive and significant association with the BMI for Non-Hispanic White children. For Non-Hispanic Black children, this positive association was reversed, although non-significant. As Figure 1 shows, we also found statistical interactions between right putamen functional connectivity to the salience network and race on the children’s BMI. These suggest that the inverse association between right putamen functional connectivity to the salience network and BMI is weaker for Non-Hispanic Black vs. Non-Hispanic White children.

### 3.5. Further Results

When we ran similar models with family structure as the moderator, the interaction between family structure and putamen functional connectivity with salience network on children’s BMI was statistically significant. Similar to race, we found positive association in married and negative association in non-married families. However, we did not find any moderating effects of parental education or household income on our association of interest. In other terms, the association between putamen functional connectivity with salience network and children’s BMI showed similar patterns in households with low and high income or across various levels of parental education. Our additional analysis showed that similar to Non-Hispanic Black children, Other/Mixed Race children also showed more negative association between putamen functional connectivity with salience network and BMI compared to Non-Hispanic White children. Finally, we found that these MDRs can be only seen for right but not left putamen resting state functional connectivity with salience network.

## 4. Discussion

There were racial variations in the association between right but not left putamen functional connectivity to the salience network and BMI of 9–10-year-old American children. While we did not find any association between right putamen functional connectivity to the salience network and BMI in the total sample of American children, interaction terms were found between race and right putamen functional connectivity to the salience network on childhood BMI, indicating weaker effects for Non-Hispanic Black (and Other/Mixed Race) children than Non-Hispanic White children. In Non-Hispanic White children, there was a positive and significant association, and in Non-Hispanic Black and in Other/Mixed Race children, there was an inverse association between right putamen functional connectivity to the salience networks and BMI. In addition, marital status also showed a similar moderating effect, however, our association was similar across subgroups based on parental education and household income. This observation suggests that while marital status may be a reason race moderates the role of putamen functional connectivity as a neural determinant of childhood BMI, parental education and household income are not the reason we see racial differences in the salience of putamen functional connectivity as a predictor of childhood BMI.

Our result on the association between right putamen functional connectivity to the salience network and BMI in Non-Hispanic White children is in line with the other research on putamen function. Putamen closely operates with the neurotransmitter dopamine and influences motivated behaviors such as food-seeking [2,3,4]. Putamen can be seen as one of the central elements of the brain’s dopaminergic system that regulates the reward seeking, including but not limited to food consumption [5].

Our second finding that putamen functional connectivity to the salience network showing weaker association with BMI for Non-Hispanic Black children than Non-Hispanic White children is an extension of the MDRs literature. Our past research using the ABCD data [26,27,39,40], Add Health [17], FFCWS [29,34,35,41,42,43,44], MTF [36], NSAL [30], FAS [45], and FACHS [47,48] have all shown significantly weaker effects of individual-level risk and protective factors (e.g., SES, age, coping, and affect) on various health outcomes for Non-Hispanic Black children in comparison with Non-Hispanic White children. For example, family income and parental education showed stronger association with aggression [31], tobacco use [33], school bonding [35], school performance [36], ADHD [29], impulsivity [34], inhibitory control [21], stress [28,41], obesity [44], physical health [31], and depression [30] for Non-Hispanic White children when compared with Non-Hispanic Black children.

The current results can be seen as Non-Hispanic Black (and Other/Mixed Race) children’s diminished salience [19] of putamen functional connectivity on BMI. MDRs are defined as systematically smaller effects of individual-level risk and protective factors such as economic resources and psychological assets for Non-Hispanic Black families in comparison with Non-Hispanic White families. As the same pattern can be seen for Latino [31], Asian American [37], and Native American [77] families, this phenomenon is believed to be due to mineralization of all marginalized social groups. These MDRs are nothing specific to Black people as they are hold for LGBT [78,79], immigrant [80,81,82], and even marginalized Non-Hispanic White families [17]. As a result, they are believed to be due to marginalization, segregation, and discrimination [17]. In our study also, we could see similar MDRs for two non-White groups of children.

A number of recent studies have documented MDRs of SES effect on several brain morphometric and functional features in the ABCD data. In one study, SES showed a weaker effect on amygdala volume of Non-Hispanic Black than Non-Hispanic White children [83]. In another study, the effect of age on amygdala and cortical function to threat was significantly different in Non-Hispanic Black and Non-Hispanic White children [84]. In several other studies, effects of SES on neurocognitive outcomes such as attention, memory, executive function, and inhibitory control were all weaker for Non-Hispanic Black than Non-Hispanic White children.

As a result of MDRs, regardless of risk or protective factors such as age, SES, coping, health, or affect, Non-Hispanic Black children remain at risk of tobacco use [33], anxiety [45], ADHD [29], depression [85], suicide [27], obesity [44], poor diet [86], high screen time [87], and low physical activity [88]. While for Non-Hispanic White children, individual level risk factors have high salience on aggression, obesity, tobacco use, and chronic disease, for Non-Hispanic Black children, individual-level risk and protective factors show diminished effects [31]. These patterns are attributed to racism, segregation, and discrimination, all reducing the relevance of individual level assets and resources on securing outcomes for the marginalized group. Our finding also showed that race moderates the salience of putamen connectivity, but not because race is a proxy of SES. This was observed as parental education and household income did not alter the salience of putamen functional connectivity as a predictor of childhood BMI.

This paper documented MDRs in Non-Hispanic Blacks as well as Other/Mixed Race children. This finding is another piece of evidence suggesting that MDRs are not specific to Blacks but can also happen in other racial and ethnic groups that are marginalized. Although most MDRs are shown for Non-Hispanic Black [19] families, similar results have been shown for Latino [31], Asian American [37], Native American [77], LGBT [78,79], immigrant [80,81], and marginalized Non-Hispanic White [17] families. This systemic nature of MDRs suggests that it is the society and not a specific social group that is to blame. In other term, it is white privilege that strengthens the salience of individual-level risk and protective factors. Potential processes that penalize the marginalized groups include social stratification, societal injustice, and contextual factors such as neighborhood poverty and school segregation, all contributing to MDRs.

Finally, we found MDRs for right but not left putamen functional connectivity. Interestingly, past research has suggested that food reward is predominantly processed in right but not left putamen and other parts of the basal ganglia. Some research suggests that money, erotic, and food rewards have different lateralizations. Our finding is in support of relevance of right but not left putamen functional connectivity to children’s BMI.

## 5. Limitations

This study only described the MDRs without exploring the underlying mechanisms behind them. A wide range of potential societal processes may cause MDRs. It is still unknown if these MDRs can be seen across all neighborhoods or require specific social and physical contexts to emerge. Residential and school segregation, neighborhood SES and crime, and environmental toxins may contribute to the observed MDRs of SES for marginalized families. There is a need to study how parents can interfere with the emergence of these MDRs in Non-Hispanic Black children. Future research may quantify how segregation, discrimination, and school quality reduce the effects of known risk factors for Non-Hispanic Black children. Finally, there is a need to study variations across geographic places. Such research may suggest public policies that can mitigate such MDRs.

## 6. Conclusions

The association between right but not left putamen’s functional connectivity to the salience network and children’s BMI depends on race and marital status but not household income and parental education. We found that putamen’s functional connectivity to the salience network is a weaker predictor of BMI for Non-Hispanic Black and Other/Mixed Race children than Non-Hispanic White children. That is, while right putamen functional connectivity to the salience network is related to children’s BMI for Non-Hispanic White children, this association is diminished for some of the Non-White racial groups. Future research should investigate the role of obesogenic environment, stress, and other factors that may keep racial minority children at risk of high BMI, regardless of putamen functional connectivity to the salience network. Residential segregation, neighborhood disorder, fast food availability, family structure, and family’s food environment may explain the weaker association of putamen functional connectivity and BMI for Non-Hispanic Black and Other/Mixed Race children.

## Figures and Tables

**Table 1:** Descriptive statistics in the sample overall and by race.

	Non-Hispanic White	Non-Hispanic Black	All
Characteristics	5180	1293	6473
	*n* (%)	*n* (%)	*n* (%)
Sex			
Female	2455 (47.4)	659 (51.0)	3114 (48.1)
Male	2725 (52.6)	634 (49.0)	3359 (51.9)
Married Family *			
No	904 (17.5)	912 (70.5)	1816 (28.1)
Yes	4276 (82.5)	381 (29.5)	4657 (71.9)
	*Mean (SD)*	*Mean (SD)*	*Mean (SD)*
Age (month) #	119.36 (7.50)	118.97 (7.24)	119.28 (7.45)
Body mass index (BMI) *	17.79 (3.19)	20.16 (4.67)	18.27 (3.66)
Right putamen functional connectivity with the salience network *	−0.01 (0.14)	0.01 (0.21)	0.00 (0.16)
Neighborhood income (USD) *	88,545.41 (32,805.06)	50,188.23 (26,596.20)	80,883.45 (35,179.56)

# *p* < 0.1, * *p* < 0.05.

**Table 2:** Model fit.

	*Model 1* Non-Hispanic White	*Model 2* Non-Hispanic Black	*Model 3* All (Main Effects)	*Model 4* All (Interaction Effects)
*N*	5180	1293	6473	6473
*R*-squared	0.03665	0.03491	0.08797	0.08932
Δ*R*-squared	0.00103	0.00232	2 × 10^−5^	0.02225
Δ*R*-squared %	0.1%	0.23%	0%	2.23%

**Table 3:** Parameter estimates for the effects of right putamen functional connectivity to the salience network on children’s body mass index (BMI).

		*Model 1* Non-Hispanic White				*Model 2* Non-Hispanic Black		
	B	SE	*p*	Sig	B	SE	*p*	Sig
Right putamen functional connectivity to the salience network	0.69357	0.29945	0.021	*	−1.02463	0.59173	0.084	#
Neighborhood income	−0.00001	0.00000	<0.001	***	−0.00001	0.00001	0.005	**
Married family	−0.91663	0.12148	<0.001	***	0.03622	0.30323	0.905	
Sex (male)	−0.14641	0.08752	0.094	#	−1.19599	0.25704	<0.001	***
Age (month)	0.04709	0.00555	<0.001	***	0.06946	0.01735	<0.001	***

# *p* < 0.1, * *p* < 0.05, ** *p* < 0.01, *** *p* < 0.001.

**Table 4:** Parameter estimates for the effects of right putamen functional connectivity to the salience network on children’s BMI.

	*Model 3* All (Main Effects)				*Model 4* All (Main Effects + Interactions)			
	B	SE	*p*	Sig	b	SE	*p*	Sig
Right putamen functional connectivity to the salience network	0.08675	0.26627	0.745		0.71048	0.33376	0.033	*
Neighborhood income	−0.00001	0.00000	<0.001	***	−0.00001	0.00000	<0.001	***
Married family	−0.66963	0.11643	<0.001	***	−0.67237	0.11634	<0.001	***
Sex (male)	−0.35512	0.08716	<0.001	***	−0.35713	0.08710	<0.001	***
Age (month)	0.05119	0.00559	<0.001	***	0.05139	0.00559	<0.001	***
Race (Non-Hispanic Black)	1.60851	0.13736	<0.001	***	1.61388	0.13726	<0.001	***
Right putamen functional connectivity to the salience network × race (Non-Hispanic Black)	-	-	-	-	−1.71156	0.55246	0.002	**

* *p* < 0.05, ** *p* < 0.01, *** *p* < 0.001.

**Figure A1:**
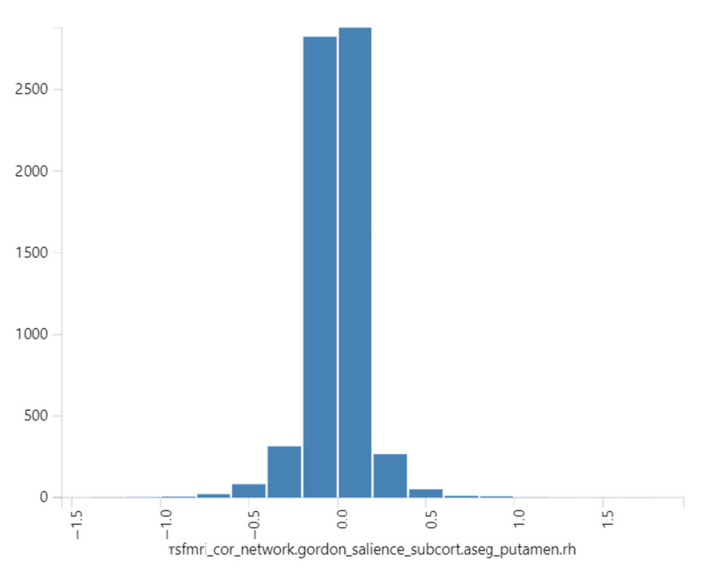
Distribution of our predictor.

**Figure A2:**
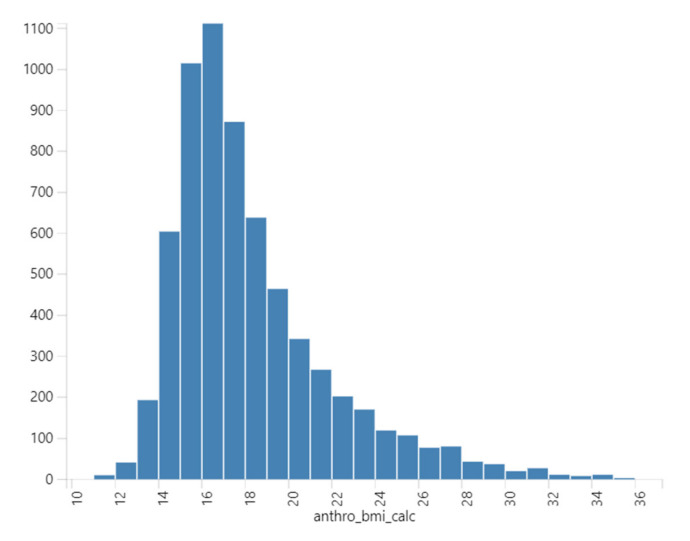
Distribution of our outcome.

**Figure A3:**
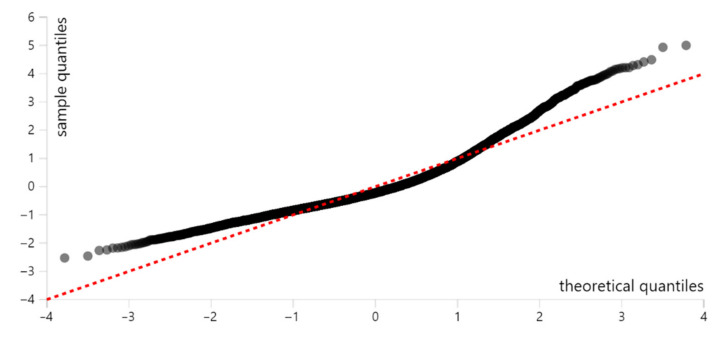
Quantiles distribution.

**Figure A4:**
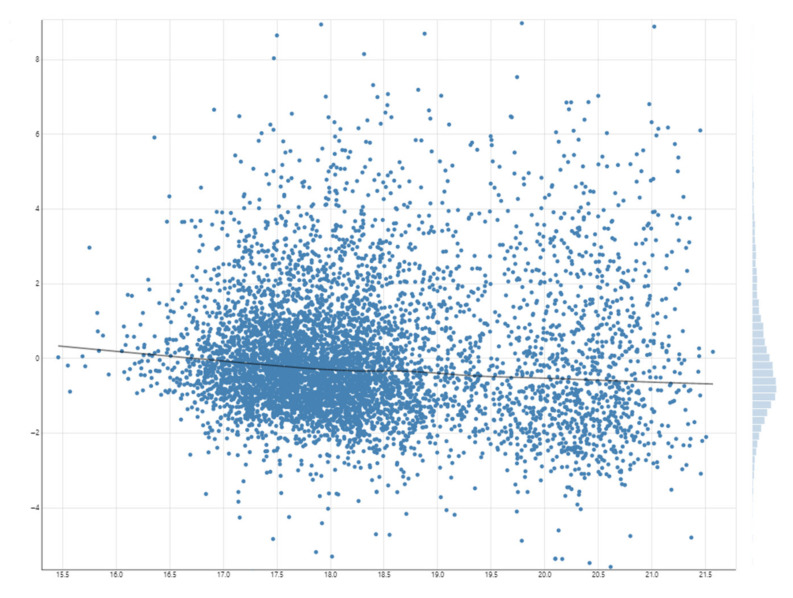
Model residuals (error terms).

## Data Availability

ABCD data are available at https://nda.nih.gov/abcd (accessed on 1 March 2021) and can be accessed after NIH approval. More information about how to access ABCD data please see https://abcdstudy.org/ (accessed on 1 March 2021) and https://nda.nih.gov/abcd (accessed on 1 March 2021).
